# Origin of the nucleus and Ran-dependent transport to safeguard ribosome biogenesis in a chimeric cell

**DOI:** 10.1186/1745-6150-3-31

**Published:** 2008-07-24

**Authors:** Gáspár Jékely

**Affiliations:** 1 Max Planck Institute for Developmental Biology, Spemannstrasse 35. 72076 Tübingen, Germany

## Abstract

**Background:**

The origin of the nucleus is a central problem about the origin of eukaryotes. The common ancestry of nuclear pore complexes (NPC) and vesicle coating complexes indicates that the nucleus evolved via the modification of a pre-existing endomembrane system. Such an autogenous scenario is cell biologically feasible, but it is not clear what were the selective or neutral mechanisms that had led to the origin of the nuclear compartment.

**Results:**

A key selective force during the autogenous origin of the nucleus could have been the need to segregate ribosome factories from the cytoplasm where ribosomal proteins (RPs) of the protomitochondrium were synthesized. After its uptake by an anuclear cell the protomitochondrium transferred several of its RP genes to the host genome. Alphaproteobacterial RPs and archaebacterial-type host ribosomes were consequently synthesized in the same cytoplasm. This could have led to the formation of chimeric ribosomes. I propose that the nucleus evolved when the host cell compartmentalised its ribosome factories and the tightly linked genome to reduce ribosome chimerism. This was achieved in successive stages by first evolving karyopherin and RanGTP dependent chaperoning of RPs, followed by the evolution of a membrane network to serve as a diffusion barrier, and finally a hydrogel sieve to ensure selective permeability at nuclear pores. Computer simulations show that a gradual segregation of cytoplasm and nucleoplasm via these steps can progressively reduce ribosome chimerism.

**Conclusion:**

Ribosome chimerism can provide a direct link between the selective forces for and the mechanisms of evolving nuclear transport and compartmentalisation. The detailed molecular scenario presented here provides a solution to the gradual evolution of nuclear compartmentalization from an anuclear stage.

**Reviewers:**

This article was reviewed by Eugene V Koonin, Martijn Huynen, Anthony M. Poole and Patrick Forterre.

## Open peer review

Reviewed by Eugene V Koonin, Martijn Huynen, Anthony M. Poole and Patrick Forterre. For the full reviews, please go to the Reviewers' comments section.

## Background

The nuclear compartment is the defining universal feature of eukaryotic cells. The recently recognized structural similarity of nuclear pore complex (NPC) components and vesicle coat complexes (including COPI, COPII and clathrin coats) indicates that NPCs and the nuclear envelope (NE) evolved by the modification of a vesicle-trafficking system [1,2]. This supports autogenous scenarios for the origin of the nucleus [3]. These models posit that the NE evolved by the incomplete fusion of pre-existing secretory endomembranes around the chromatin of a protoeukaryotic cell [4], and not via endosymbiosis [5].

Unlike e.g. the ER or peroxisomes, the nucleus is not a topologically separated compartment in the eukaryotic cytoplasm since the nucleoplasm and the cytoplasm are continuous through the nuclear pores. Yet, the nucleus has a distinct composition due to the directionality and selectivity of nuclear transport. This is achieved by the Ran GTPase cycle, selective cargo binding by karyopherins, and selective permeability of NPCs [6-8]. The nucleus is organized around chromatin through the action of the Ran GTPase system that is universally present in eukaryotes [9,10]. Eukaryotes evolved the Ran system very early during their evolution to mark the position of chromatin. The evolution of the Ran system had key importance during the origin of nuclear compartmentalisation given the universal and fundamental roles of Ran in several aspects of nuclear function, including nuclear transport [11], NPC assembly [12], NE assembly [13], kinetochore function [14] and mitosis [15]. Ran evolved from other membrane-trafficking small GTPases that all act as molecular switches and orchestrate downstream molecular events by binding to effector molecules in their GTP-bound form [16]. RanGTP could have initially regulated membrane traffic or a primitive anuclear form of mitosis [9]. Without Ran and a directional and selective nuclear transport the nucleus would not exist. To understand the origin of nuclear compartmentalisation we therefore have to understand the co-evolution of nuclear identity (Ran system), nuclear membranes, and selective nuclear transport.

Recently, it has been proposed that the uptake of the precursor of mitochondria, an alphaproteobacterium triggered nuclear compartmentalisation by infecting a host archaebacterial cell with type II introns [17]. Although this model is attractive, objections can be made why uncontrolled intron spread could not have happened in an archaebacterial host cell without meiotic sex [18]. The intron spread model also fails to account for the origin of selective nuclear transport.

Here I propose a model that explains the origin of all three key elements of nuclear compartmentalisation, i.e. nuclear identity, nuclear membranes, and selective nuclear transport. I argue that the protoeukaryotic lineage, originating as a sister group to crown archaebacteria [4,19], evolved a nucleus to compartmentalise its ribosome factories. This was vital to prevent chimerism between host ribosomes and host-encoded RPs of the protomitochondrium. Efficient segregation could only be achieved by the evolution of the Ran system to spatially mark chromatin, of karyopherins to chaperon RPs, and of selective valves (the NPCs) to regulate nuclear permeability. This model proposes a causal link between the evolution of the nuclear compartment and the acquisition of the mitochondrial ancestor, in agreement with the intron-spread model. Ribosome chimerism, however, also provides a direct link between the selective forces driving compartmentalisation and the evolution of the cellular mechanisms achieving it.

## Results and discussion

### Chimeric ribosome formation in an anuclear protoeukaryotic cell

Mitochondria originated before the radiation of eukaryotes given that all extant eukaryotes have or their ancestors once had mitochondria [20-24]. It is also clear that extensive gene transfer from the protomitochondrial symbiont to the host cell's genome during the early stages of symbiosis made a major contribution to the origin of eukaryotes [25]. Whether mitochondria originated during a stage where the host cell was still anuclear cannot be ascertained based on comparative genomic, phylogenetic or other evidence. However, the phyletic distribution of some NPC and nuclear transport cycle components suggests that they are of probable symbiotic origin [10]. This would mean that the acquisition of mitochondria overlapped with or predated the evolution of the nucleus.

**Figure 1:**
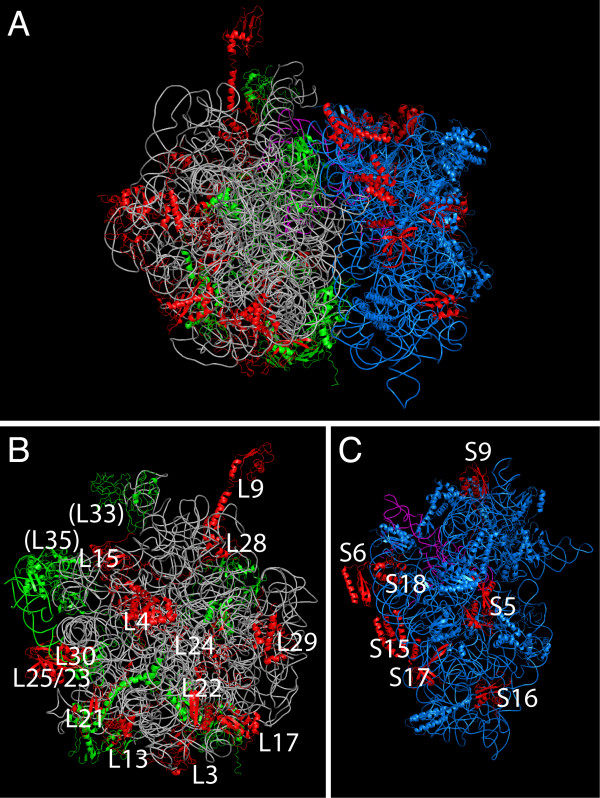
**Ribosome chimerism in the protoeukaryotic cell**. RPs that were transferred to the host genome in the eukaryotic stem lineage are highlighted in red in the structure of the *Thermus thermophilus *70S ribosome (A) large (B) and small (C) subunits (PDB codes 2J03 and 2J02). The small subunit 16S RNA and proteins are coloured in blue, 23S RNA in grey, large subunit proteins in green, A-, P- and E-site tRNAs in magenta. The ancestrally host-encoded proteins are labelled, L33 and L35 are on the other side of the structure.

The present model starts with an anuclear protoeukaryote that already possessed an endomembrane system and acquired the protomitochondrium. The symbiont started to be converted into an organelle by the processes of endosymbiotic gene transfer and host control. A survey of all mitochondrial genomes indicates that the genes that were transferred to the host genome in the period before the radiation of crown eukaryotes (i.e. in the stem eukaryotic lineage) also included 24 genes encoding mitochondrial-RPs (L3, L4, L7/12, L9, L13, L15, L17, L21, L22, L24, L25/23, L28, L29, L30, L33, L35, S5, S6, S9, S15, S16, S17, S18, S21). The transfer of ribosomal genes at an anuclear stage would have meant that mitochondrial-RPs were synthesized in the same cytoplasmic compartment where host ribosomes were synthesized and assembled [4]. The two types of RPs (archaebacterial/eukaryotic type of the host and eubacterial type of the protomitochondrium) can form chimeric ribosomes, as shown by biochemical studies of extant eu- and archaebacteria ribosomes [26-30]. Mapping the 24 ancestrally host-encoded mitochondrial-RPs to the structure of the 70S ribosome [31] highlights the scale of the problem (Figure 1). The potential incorporation of up to 24 different mitochondrial-RPs instead of host-RPs upon subunit assembly could have produced chimeric ribosomes with reduced or no function. The suboptimal functioning of chimeric ribosomes is supported by the rarity of evolutionary gene replacement between cytoplasmic and organellar RPs [32], as opposed to metabolic enzymes [33] or tRNA synthases [34]. The chimeric cell therefore faced the problem of distinguishing and sorting two sets of RPs that were synthesized in the same compartment.

### A RanGTP-dependent karyopherin system can reduce chimerism at an anuclear stage

The ribosome chimera load, resulting from the fixation of slightly deleterious individual RP gene transfers from symbiont to host created selective pressure to reduce chimerism. Compensatory advantageous mutations that reduced chimerism and restored fitness started to spread. The back-transfer of mitochondrial-RP genes after the loss of the original mitochondrial copy and the acquisition of mitochondrial targeting signals was not possible. This unidirectionality (gene transfer ratchet) stems from the fact that whereas mitochondrial genes can integrate into the host genome following lysis of mitochondria, there is no mechanism for reverse transfer. As shown below, invoking a selective pressure to reduce ribosome chimerism, the model can explain key steps in the origin of nuclear compartmentalisation.

In the first evolutionary stage described by the model, the protoeukaryotic cell was still anuclear. The genome was attached to an evolving endomembrane system [4,9]. Host ribosomes were assembled around chromatin because assembly was seeded on the transcribing host rRNAs. Mitochondrial-RPs were synthesized in the cytoplasm and could freely diffuse to the chromatin region and incorporate into host ribosomes, leading to the formation of chimeric ribosomes. Ribosome chimerism was reduced when the cell evolved mechanisms to enrich host-RPs around chromatin. This involved the evolution of the Ran system as a chromatin mark and its employment to influence the distribution of free RPs in the cell. Ran evolved its ability to mark the position of chromatin when its exchange factor (RCC1) acquired chromatin localization. The localized GDP to GTP exchange created a region in the cytoplasm with higher RanGTP concentration. The chromatin-enriched RanGTP could reduce ribosome chimerism. It is sufficient to invoke a chaperone system binding to free RPs and that RanGTP regulated these RP-chaperon complexes. When RanGTP could selectively dissociate host-RP-chaperon complexes but not mitochondrial-RP-chaperon complexes, host-RPs enriched around chromatin. These ribosomal chaperons (hereafter called karyopherins) are conceived as the ancestors of the karyopherin family of nuclear transport receptors that still retain chaperon activity [35].

**Figure 2:**
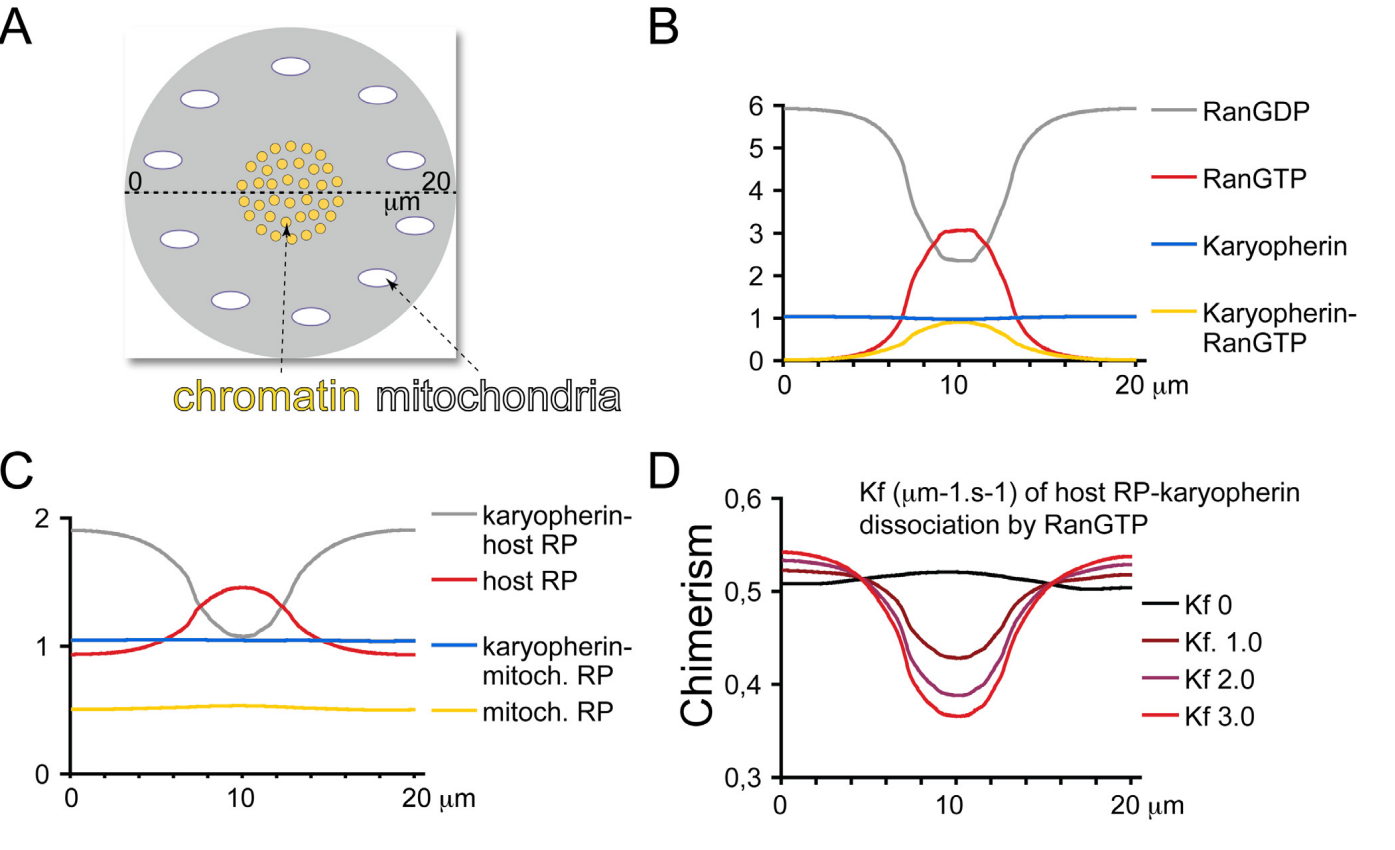
**RanGTP dependent karyopherin-RP dissociation reduces chimerism**. (A) The geometry used for the simulations has no NE, chromosomes and mitochondria are in the cytoplasm. (B) Steady state distribution of RanGTP, RanGDP, karyopherin and karyopherin-RanGTP along the line shown in panel (A). (C) Steady state distribution of host-RP, mitochondrial-RP, karyopherin-host-RP and karyopherin-mitochondrial-RP complexes along the line shown in panel (A). (D) Chimerism (mitochondrial-RP/host-RP) at different values of the forward rate constant for the RanGTP driven host-RP-karyopherin dissociation reaction (host-RP-karyopherin + RanGTP => host-RP + karyopherin-RanGTP) along the line shown in panel (A).

Computer simulations using the program Virtual Cell with a spatial reaction-diffusion model show that an increasing influence of RanGTP on karyopherin-RP dissociation can progressively reduce chimera load (Figure 2). The spatial model used in the simulations has a cytoplasmic domain with a central chromatin region and peripheral mitochondria (Figure 2A). RCC1, the exchange factor of Ran, is localized to the surface of chromatin and is not diffusible. The reaction-diffusion system consisting of localized GDP to GTP exchange and uniform GTP hydrolysis by Ran (stimulated by RanGAP) creates a RanGTP gradient with its maximum around chromatin (Figure 2B). This RanGTP gradient can then influence the distribution of RPs. One host and one mitochondrial-RP were modelled, both bound by a uniformly distributed karyopherin. Due to the dissociation of host-RP-karyopherin complexes by RanGTP, host-RPs enrich around chromatin (Figure 2C). This results in the local decrease in mitochondrial-RP to host-RP ratio, which was used as a measure of chimerism (Figure 2D). The evolution of this RanGTP driven host-RP-karyopherin dissociation (RanGTP + host-RP-karyopherin => RanGTP-karyopherin + host-RP) could have efficiently reduced chimerism as shown with four simulations using an increasing forward rate constant for the reaction (Figure 2D). These results show that an emerging RanGTP-karyopherin system could have reduced ribosome chimerism even without a NE and NPCs.

### An open NE limits diffusion and sharpens the RanGTP gradient

**Figure 3:**
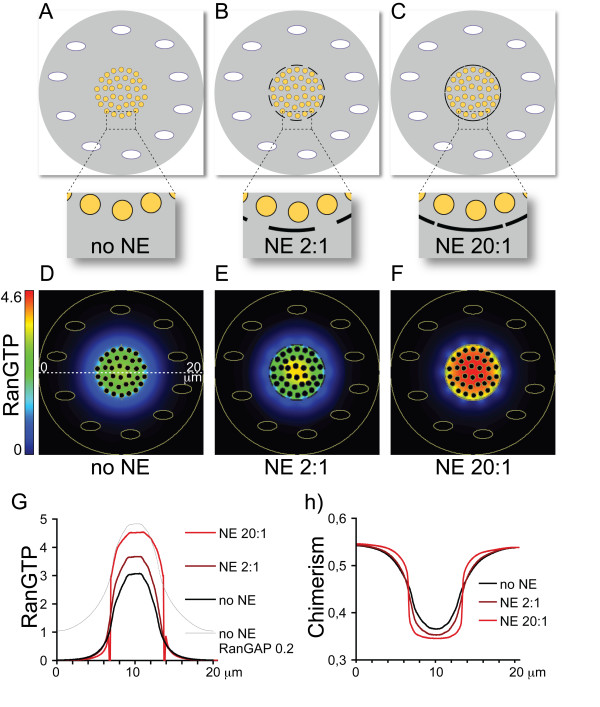
**An open membrane network sharpens the RanGTP gradient and reduces ribosome chimerism**. The three geometries used for the simulations represent intermediate evolutionary stages with progressively more membranes around chromatin. The ratio of the size of the membrane domains and the gaps between them increases from no membranes (no NE, panel A), through 2:1 (B), to 20:1 (C). Although the chromosomes are embedded in a membrane network, there is no selective nuclear transport. Every component can freely diffuse across the gaps between the membrane domains. In the simulations all parameters, except for the geometry, were identical. (D-F) Steady state distribution of RanGTP in the three geometries. (G) Steady state distribution of RanGTP in the three geometries along the line shown in panel (D). The grey line shows the effect in the no NE geometry of decreasing RanGAP levels from 1 to 0.2 um. (H) Chimerism (mitochondrial-RP/host-RP) at steady state with the three different geometries. The black curve (no NE) is the same as the red curve on Figure 2D) (Kf 3.0).

The next key step in the evolution of nuclear compartmentalisation was the development of a membrane network around chromatin from the secretory endomembrane system of the protoeukaryote. The evolution of the NE from pre-existing, dynamic endomembranes is strongly supported by the evolutionary relationship between NPC components and vesicle coat complexes [1,2]. If the NE evolved from intracellular membranes (and not e.g. from a symbiont's membrane), as these studies strongly suggest, then there was necessarily a stage with an open NE surrounding chromatin, without well developed NPCs. Clearly such an open membrane network without NPCs could not have maintained selective nuclear transport and therefore a compositionally distinct nuclear compartment. On the other hand, NPCs embedded in the NE, could only have evolved when a membrane network was already present around chromatin. This problem can only be resolved by supposing that a primordial, open NE initially evolved around chromatin for reasons other than supporting NPC-dependent transport. Here I suggest that the primordial open NE initially served as a diffusion barrier that created a sharper RanGTP gradient. This contributed to the definition of the evolving nucleoplasm and further reduced ribosome chimerism. Virtual Cell simulations using identical parameters but three different geometries (Figure 3A–C) show that an open membrane system increases RanGTP concentration around chromatin and sharpens the RanGTP gradient (Figure 3D–G). The higher RanGTP levels increase the localised release of host-RPs from karyopherin and further reduce the chimera load (Figure 3H). Furthermore, the sharper RanGTP distribution also sharpens the distribution of host-RPs and makes it more uniform in the ribosome factories.

Importantly, the effect of an open NE on RanGTP distribution is not equivalent to generally increasing RanGTP levels in the cell. This could be achieved e.g. by reducing RanGAP levels (from 1 to 0.2 μm, Figure 3G) and thereby GTP hydrolysis by Ran. This would lead to an increase in RanGTP levels also in the non-chromatin regions of the cytoplasm and would blur the distinction between chromatin and non-chromatin regions of the cell (Figure 3G). An open membrane network around chromatin on the contrary leads to a local increase in RanGTP concentration and therefore the further differentiation of cytoplasm and nucleoplasm. This diffusional effect on RanGTP distribution, even without selective nuclear transport, is the likely selective cause for the evolution of a dense membrane network around chromatin. This membrane network subsequently served as the scaffold for the formation of NPCs.

### Evolution of a selective hydrogel sieve at nuclear pores

The evolution of a dense membrane network was the precondition for the evolution of NPCs and selective nuclear transport. NPCs evolved by the modification of membrane coating complexes of the developing eukaryotic endomembrane system [1]. These complexes initially probably functioned in shaping the open membrane network around chromatin and in preventing its complete fusion [4]. Selectively permeable NPCs evolved as these coat complexes developed a meshwork of natively unfolded FG repeats in the holes of the *n*-torus (where *n *is the number of pores). FG nucleoporins can evolve quickly [36] and repeat expansion could have rapidly created a dense meshwork of FG repeats during the origin of NPCs. This meshwork further limited diffusion between the two sides of the NE. As the FG network started to form crosslinks and became a hydrogel [37,38], it was no longer permeable to larger molecules. Only transport carriers (karyopherins) and cargo bound to them evolved the capacity to selectively permeate the pores. RanGTP dissociated the karyopherin-cargo complexes that traversed the pores at the nuclear side.

As NPCs progressively closed up, other nuclear cargos also had to be imported specifically. These secondary cargo proteins evolved nuclear localization signals (NLS) that mimicked the sequences used by karyopherins to recognize host-RPs. Given that RPs are the most basic proteins in the cell, some of these recognition sequences could have been short basic stretches of amino acids generally occurring in RPs. These motifs had to be refined to allow specific recognition of individual host-RPs, alternatively, some host-RPs evolved novel regions for karyopherin binding. These include for example L25 that has an extra N-terminal NLS-carrying region, absent from archaebacterial homologues [39]. Some other cargoes, including DNA-binding proteins, could have easily evolved NLSs by the slight modification of their basic DNA-binding regions.

At this stage several processes had to evolve simultaneously. Nuclear export pathways evolved in parallel with import pathways. Following gene duplications two karyopherin types evolved, one for import and one for export. Contrary to import karyopherins, the export karyopherins bound cargo in the presence of RanGTP. The RanGTP gradient was further sharpened when RanGAP became predominantly cytoplasmic by acquiring export signals. Further differentiation of cytoplasm and nucleoplasm occurred when the assembly of 80S ribosomes was prevented in the nucleus and the large and small subunits were exported separately using a Crm1 and RanGTP-dependent mechanism [40,41]. This restricted protein synthesis to the cytoplasmic side.

**Figure 4:**
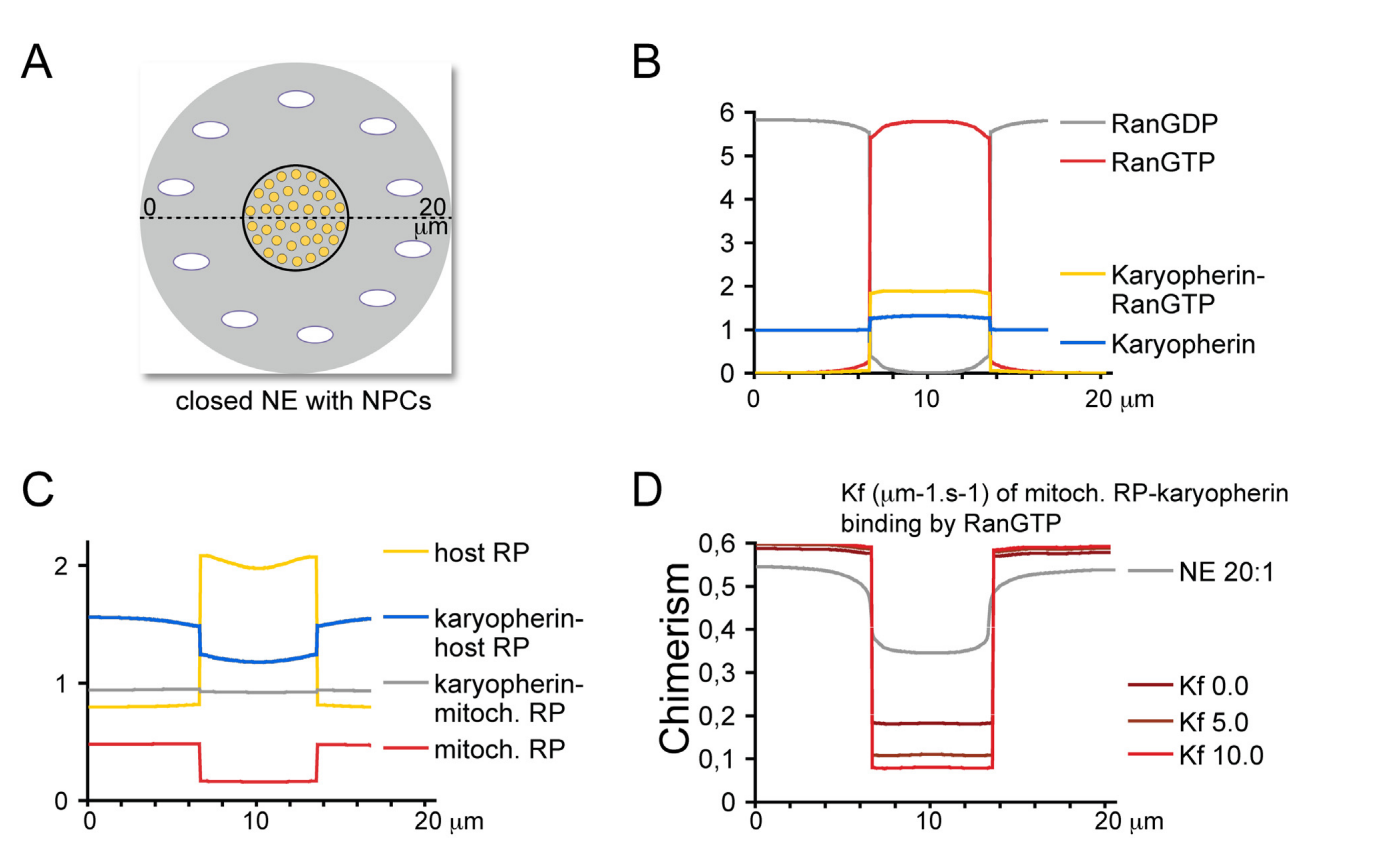
**Selective nuclear transport segregates the cytoplasm and nucleoplasm**. The geometry used for the simulations has a closed NE (A). Transport via NPCs is defined as fluxes across the NE. (B) Steady state distribution of RanGTP, RanGDP, karyopherin and karyopherin-RanGTP along the line shown in panel (A). (C) Steady state distribution of host-RP, mitochondrial-RP, karyopherin-host-RP and karyopherin-mitochondrial-RP complexes along the line shown in panel (A). (D) Chimerism (mitochondrial-RP/host-RP) at different values of the forward rate constant for the mitochondrial-RP, karyopherin-RanGTP exchange reaction (mitochondrial-RP + karyopherin-RanGTP => mitochondrial-RP-karyopherin + RanGTP) along the line shown in panel (A). The grey curve (NE 20:1) is the same as the red curve on Figure 3H (NE 20:1), and is shown for comparison.

In the final stage that was modelled, the NE was closed and transport across NPCs was described as fluxes. RanGAP was cytoplasmic and RPs were only synthesized in the cytoplasm (see Figure 4). RPs could only cross the NE when bound to karyopherin (this disregards passive transport of small RPs that can happen but is less efficient then karyopherin-dependent transport). RanGTP dissociated host-RP-karyopherin complexes and stabilized mitochondrial-RP-karyopherin ones. Ribosome chimerism was greatly reduced compared to the open NE stages. At this stage, which is similar to the situation in modern eukaryotes, the nucleus is fully defined compositionally.

The ribosome chimerism model presents a detailed and gradual molecular scenario on how a compositionally distinct nuclear compartment could have evolved. It can account for several independent factors and their role in the evolution of compartmentalisation. These factors include the parameters of the RanGDP/GTP cycle, the geometry of the endomembrane system and the permeability of NPCs. I proposed ribosome chimerism as the likely selective factor driving these changes, although it was probably not the exclusive one.

The model agrees with the intron-spread model [17] in some important aspects but disagrees in others. Both models agree that the acquisition of the protomitochondrium and ensuing endosymbiotic gene transfer were the primary causes triggering the evolution of nuclear compartmentalisation. Whether the host that acquired the protomitochondrium was phagotrophic or not, although a major unresolved debate [4,24,42-45], is not strictly relevant for any of the models. The main difference is that the current model does not require the spread of spliceosomal introns before the origin of nuclear compartmentalisation. It is more consistent with the idea that introns started to spread selfishly only after the separation of transcription and translation [46] and that this spread was facilitated by the origin of intron-dependent nonsense-mediated mRNA decay [47].

## Conclusion

Two important factors can be distinguished in the evolution of the nucleus, a neutral and a selective one. The fixation of slightly deleterious unidirectional transfer events of protomitochondrial genes to the host genome (gene transfer ratchet) was neutral and resulted in chimerism and reduced fitness. The second factor was the selective spread of compensatory advantageous mutations that reduced chimerism by segregating the mixed components. I defined key cellular and molecular stages and showed a gradual transition through them during which chimerism was progressively reduced. Most importantly, it was shown that it is possible to de-couple the origin of the NE and NPCs by invoking an open NE stage where the membranes served only as a diffusion barrier.

The idea that the nucleus evolved as a protective compartment, a solution to segregate ribosome factories in an endosymbiont-carrying cell does not require foresight from evolution. This is in contrast to the frequently encountered formulation that the nucleus evolved to allow a more sophisticated control of gene regulation. Eukaryotic gene regulation, RNA processing and other nuclear functions probably only evolved secondarily, as a means to adapt to the new situation resulting from the compartmentalisation of ribosome biogenesis.

## Methods

Mitochondrial genomes were searched for the presence of RPs at the Organelle Genome Database (Gobase; ). The RPs that do not occur in any mitochondrial genomes were visualized in the structure of the *Thermus thermophilus *70S ribosome (PDB Codes: 2J02 and 2J03) using Pymol .

The simulations were run using Virtual Cell 4.4 at . The geometry size was 20 × 20 microns, the mesh size 300 × 300 points. The geometries had five volume domains (extracellular, cytoplasm, mitochondria, NE and chromatin) and four surface (membrane) domains (plasmamembrane, mitochondrial membrane, chromatin surface and NE membrane). No reactions happened in the extracellular, chromatin and NE domains. The mitochondrial membrane had a flux of mitochondrial-RP (0.5 * Mitochondrial_RP_Cytoplasm μM*μm^2^/sec) and the chromatin surface contained RCC1. The five geometries used differed only in the size of the NE membranes. Simulations were run until steady state (150 sec total time, 0.0010 sec time steps) using the finite volume method. The reactions and parameters used for the simulations are partly based on previous studies [7,48,49]. The RCC1-stimulated RanGDP/GTP exchange was modelled using Michaelis-Menten kinetics with Km = 0.5 μm and Vmax = 10.0*RCC1 molecules*μm^-2 ^sec^-1^, instead of a four-step model as in [7,48,49]. RanGAP-stimulated GTP hydrolysis by Ran was modelled using Michaelis-Menten kinetics with Km = 0.7 μm and Vmax = 10.6*RanGAP μM sec^-1^. The binding of karyopherin to host-RP and mitochondrial-RP was described with mass action kinetics with Kf = 10 μM^-1 ^sec^-1 ^and Kr = 5 sec^-1^. The dissociation of host-RP-karyopherin complexes by RanGTP was described with mass action with a Kf = 3 μM^-1 ^sec^-1^. The diffusion coefficients used were 22 μm^2 ^sec^-1 ^for RanGDP/GTP and RanGAP, 14 μm^2 ^sec^-1 ^for karyopherin, 20 μm^2 ^sec^-1 ^for RPs, and 13 μm^2 ^sec^-1 ^for karyopherin-RanGTP, karyopherin-host-RP and karyopherin-mitochondrial-RP complexes. The initial concentrations were 6 μM for Ran, 4 μM for karyopherin, 1 μM for RanGAP and 500 μM for RCC1 on the chromatin surface. Host-RP and mitochondrial-RP were synthesized with a reaction rate of 0.1 μM sec^-1 ^and decayed following mass action with 0.1*RP mM sec^-1^.

The models are deposited into the public model database of Virtual Cell at .

## Competing interests

The author declares that he has no competing interests.

## Reviewers' comments

### Reviewer's report 1

Eugene V Koonin National Center for Biotechnology Information, National Library of Medicine, National Institutes of Health, Bethesda, MD 20894, USA

The driving force behind the emergence of the nucleus is a key aspect of eukaryogenesis. In this paper, Jékely proposes a new idea, namely, that the evolution of the nucleus from the endomembrane system was driven primarily by the necessity to avoid the formation of hybrid ribosomes containing partly host and partly mitochondrial r-proteins. In my opinion, the general premises of this paper are correct. Indeed, there is every reason to believe, firstly, that the origin of the nucleus postdates mitochondrial endosymbiosis and, secondly, that the nuclear envelope evolved from the endomembrane system. It stands to reason, then, that the nucleus evolved under the pressure of selection for partitioning the genome from the cytosol, and that pressure was caused by the need to alleviate conflicts caused by the collision of host and mitochondrial components. I am inclined to think that, beyond this general connection, it might be futile to search for a single cause behind the origin of the nucleus. From my viewpoint, it is more reasonable to accept that multiple host-symbiont conflicts were at play. Avoidance of ribosomal chimerism, indeed, could be one of these, but so would be control of the damage caused by intron invasion, and there well could be multiple other factors.

#### Author's response

I agree that probably multiple factors played a role during the evolution of the nucleus. The role of RP chimerism is featured here prominently because the cell biological scenario that can be built on this premise is parsimonious and powerful as it provides a direct link between the source of the conflict (chimerism) and the mechanism to resolve it (nuclear transport). Regarding the role of introns, I am more inclined to think that they started to spread after, rather than before, the segregation of cytoplasm and nucleoplasm.

### Reviewer's report 2

Martijn Huynen, Nijmegen Center for Molecular Life Sciences & Center for Molecular and Biomolecular Informatics, Nijmegen, Netherlands

Gáspár Jékely provides an interesting and original rationale for the origin of the nucleus, that, in one go, explains the assembly of ribosomal complexes in the nucleus. The lynchpin of the argument is the undesirable formation of ribosome chimeras upon the transfer of mitochondrial ribosome genes to the nucleus. Upon reading the title and abstract I was a bit sceptical about the likelihood of chimeric ribosomes, but apparently these has been documented. The supporting of the hypothesis with an elegant mathematical model is appreciated, as such models often reveal holes in hypotheses. In this case this appears not to be the case. Still I have some issues with the argument that I would like the author to address:

Is there, in the literature, replacement (chimerism) in competitive situations? And connected to this question, how is the likelihood of forming chimeras in the model calculated? Is that, as I understand from the legend of the figures, the ratio of the mitochondrial RP over the host RP? How would the model behave if the likelihood of chimera's was smaller than that. These ribosomes have after all, evolved for a long time independently.

#### Author's response

Replacement could also be demonstrated in a competitive situation. In one paper [28] the endogenous *E. coli *L2 is replaced in vivo by overexpressed *Haloarcula marismortui *L2 in 25% of the ribosomes. In the model chimerism is characterised with the ratio of mitochondrial-RP over host-RP. This is an arbitrary measure, and the probability of chimera formation will affect this. If it was 10 times smaller, then the figures would also be 10 times smaller, however, the relative difference under the different condition (e.g. following the development of NE vesicles) would be the same. So irrespective of the kinetics of chimera formation, the number of chimeric ribosomes would always be proportional to the ratio of mitochondrial-RP over host-RP (unless of course the kinetic parameters of chimera formation themselves change, e.g. due to sequence divergence – see below).

We know that most of the catalytic activity in ribosomes is in the RNA, so why would replacement lead to loss of activity?

#### Author's response

I changed activity to 'function'. RPs can have a role for example in tRNA binding or downstream processes. SSU S9 for example contacts the anticodon stem-loop of P-site tRNA and LSU L23 provides a major binding site for the signal recognition particle and the translocon. One can imagine that these and other functions were less efficient in chimeric ribosomes.

If the transfer of mitochondrial ribosome genes to the nucleus leads, via the production of chimeras, to a reduced fitness, one would not expect it to occur.

#### Author's response

This point was also brought up by Patrick Forterre. The answer is that slightly deleterious individual transfers could have reached fixation by genetic drift. Once mitochondrial re-targeting evolved and the original copy was lost, there was no way back, because there is no mechanism for reverse transfer (gene transfer ratchet). The mutations that were driving compartmentalisation spread as compensatory advantageous mutations, reducing chimerism. The attractiveness of this model is that the level of fitness reduction is 'tuneable' given that up to 24 individual RP gene transfers occurred. The fixation of each transfer could have lead to fitness reduction that was compensated during the successive stages of nuclear compartmentalisation.

If chimera's were an issue, one expects the evolution of ribosomal proteins that do not form chimera's: i.e. evolution at the level of the ribosome genes to prevent "cross-hybridizations". Rather than this, the model proposes an evolution of a specificity of the RanGTP for host-RP-chaperon complexes.

#### Author's response

Sequence divergence may have been an important factor as well, as proposed by Cavalier-Smith [4]. The model requires that such divergence was not sufficient and complementary surfaces still remained that formed chimeric ribosomes.

Do I understand from the figures correctly that there is still a lot of chimerism in the cytoplasm?

#### Author's response

Yes, but exchange in assembled ribosome does not happen, or is very slow for most RPs [50]. What is critical is the mitochondrial-RP/host-RP ratio during subunit maturation. This means that nucleoplasmic chimerism was a much more serious problem for the cell than cytoplasmic chimerism.

The total number of mitochondrial ribosomal proteins of (alpha-proteo)bacterial origin now totals 53 including RPS3 and RPL29 that are homologous to MRPS24 and MRPL47 (Smits, Smeitink, van den Heuvel, Huynen and Ettema, NAR 2007). I do not know which ones of these are never found on mitochondrial genomes.

#### Author's response

RPS3 can be found in plant mitochondrial genomes but RPL29 not, so it also must have been transferred early. I included it into the list.

The difference between orange and yellow in Figure 2B and 2C is quite subtle, could another color be used?

#### Author's response

I changed the colour.

### Reviewer's report 3

Anthony M. Poole Arrhenius Laboratories for Natural Sciences, Stockholm University, Department of Molecular Biology & Functional Genomics, Stockholm, Sweden

This article proposes that the origin of the nucleus serve to reduce or eliminate the risk of ribosome chimerism arising from the transfer of mitochondrion-derived ribosomal protein genes to the host genome. I found this paper very interesting, and was particularly impressed by the author's attempt to test aspects of this hypothesis using a virtual cell model.

One key point that the reader must first accept in order to entertain the model is that the mitochondrion predates the nucleus in evolution. Obviously this viewpoint is shared by other authors (e.g. ref. [17]) but, to my mind, there is no strong evidence to support this contention. The only argument that Jékely raises to support this viewpoint is a reference to Mans et al. (ref. [10]), where it is suggested that some components of the NPC are of α-proteobacterial origin. In that paper, which is based on sequence similarities and distributions, it is tacitly assumed that NPC components must ultimately be of prokaryotic origin, and hence any similarities are interpreted as confirming this. The sequences of these proteins are sufficiently divergent as to make it impractical to address these proposed origins with phylogenetic analyses (as those authors point out). However, none of the proteins or folds of putatively α-proteobacterial origin are restricted solely to this group of bacteria (see their Table 1). I bring this point up in order to make it clear that the data used to support this order of events are weak. To my mind, it is not currently possible to ascertain whether the nucleus evolved before or after the mitochondrial ancestor was engulfed. I am nevertheless happy to concede Jékely's version of events as a possibility.

#### Author's response

I agree with this and find the comparative genomic evidence week. I modified the relevant part of the text to make this clear.

Assuming then that the mitochondrion predates the nucleus, the idea of avoidance of chimerism through intranuclear assembly of the ribosome is an attractive one. As is well known, ribosomes are not assembled in the cytoplasm but in the nucleolus, well away from the site of translation – of both host- and mitochondrion-derived ribosomal proteins. Secondly, there is of course a system for the recognition (transit peptides) and transport of mitochondrial proteins to that organelle. The outcome of these intracellular modes of molecular transport is that the two sets of proteins are kept distinct, which is what the simulation serves to show for the nuclear-targeted proteins.

However, one question that is nagging me is how much of an issue chimerism really was. Clearly, for an organelle-to-host genome gene transfer event to be fixed, there must be an existing mechanism for transport of proteins from the cytoplasm to the organelle. This sort of makes me wonder if this isn't host-initiated. That is to say, the first instances of protein targeting to the mitochondrion might in fact have been of host-encoded proteins (perhaps that served to better maintain the symbiosis in some way). This would make it somewhat easier to explain the emergence of the mitochondrial-targeting system – as is well-established, plenty of proteins of non-endosymbiont origin are now targeted to the mitochondrion. The reason I bring this up is that I can't imagine successful transfer of a mitochondrial-origin gene (meaning that the mitochondrial copy is lost), where that gene is still required to function in the mitochondrion, unless there is a pre-existing *functional *transport system to get the product back into the organelle. That suggests that the mitochondrial targeting pathway was already functioning, which would in turn reduce any chimerism simply due to the fact that these proteins would already be specifically targeted to the mitochondrion.

#### Author's response

Clearly, a mitochondrial targeting system had to be in place and mitochondrial RPs had to acquire the necessary targeting signals before the mitochondrial gene copies could be lost. However, mitochondrial RPs were still synthesized in the cytoplasm, which means that there had been a dwell time and some diffusion in the cytoplasm before the targeting signals were recognised. The simulations try to approximate such a situation, mitochondria serve as a 'sink' and mitochondrial RP import is modelled as a flux.

One further concern I have along these same lines is regarding nuclear localisation signals. In the section, 'Evolution of a selective hydrogel sieve at nuclear pores', the statement is made that nuclear localization signals 'mimicked the sequences used by karyopherins to recognise host-RPs'. The argument is then made that these could have been basic regions, which RPs have no shortage of. What bothers me about this is that this would apply equally to mitochondrial-RPs. All the more reason to assume that mitochondrial import pathways cannot be ignored, and would have been at least as important in eliminating potential chimerism. I know of a couple of references that indicate how noisy the nuclear import pathway is. One is an odd experiment (in that I am not sure why the authors did it) where nuclear localisation of an archaeal proteasome subunit is demonstrated (Nederlof et al. 1995 PNAS 92, 12060–12064). Second, a paper by Cokol et al. (EMBO Rep 1:411–415, 2000) points out that for DNA binding proteins with a known NLS, 90% of the time these two motifs overlap. Of relevance to chimerism, they go on to show that, on sequence alone, *E. coli *is estimated to produce 54 proteins that carry potential NLSs (about half of which are DNA binding motifs). If preventing mitochondrial-origin proteins from spuriously entering the nucleus were solely the domain of the nuclear transport system, I doubt it would be very effective.

#### Author's response

The template role of DNA binding proteins in the evolution of NLSs is another possibility. In this model I suggest a primary role for RP import. I would of course be interested in discussing models that explain why the cell started to bind and transport its DNA binding proteins. So far I haven't heard of one, and I couldn't come up with one either. I rather suspect, given the noisy nature of nuclear transport, that DNA binding proteins started to be transported after or along with RPs with minimal or no modification of their basic motifs. Eubacterial ribosomal proteins are also imported into eukaryotic nuclei [51].

I agree that that it is difficult, based on linear basic motifs, to segregate mitochondrial-RPs and host-RPs. The evolution of more specific recognition would be required for that. I also agree that the increased efficiency of mitochondrial targeting could have played a role as well. Additionaly, I suggested that the active exclusion of mitochondrial-RPs by export mechanisms could have played a role. I didn't find experimental evidence in favour of this possibility though.

None of this is to say that the theory rendered implausible – it is not. However, it is a shortcoming of the current paper that it does not consider the machinery for mitochondrial-targeting.

Another question I have is how the nucleolus ties into all this. Given that this organelle maintains its integrity within the nucleus despite not being membrane-bound, and is the site of rRNA transcription and ribosome subunit assembly, is it really necessary to invoke the nuclear envelope to explain reduced chimerism? If the author has any comments on this, it would be interesting to hear them.

#### Author's response

I don't think that the nucleolus can serve as a specific diffusion or permeability barrier for certain proteins, as the NE and NPCs can, even though it has its integrity.

Finally, a comment. While this type of paper typically stands or falls on whether the primary argument is accepted, there are some additional, important, and uncontroversial insights on the stepwise origin of certain features of the nuclear pore complex and nuclear transport that are important contributions in themselves. Specifically, the section 'Evolution of a selective hydrogel sieve at nuclear pores' illustrates how recent experimental results [37,38] shed light on the stepwise evolution of nuclear pore selectivity. Likewise, the importance of RanGTP and karyopherins to our understanding of the evolution of the emergence of a fully-fledged nuclear transport system should be clear after reading this paper.

#### Author's response

I am happy to read this comment. My selective scenario may not be correct, but the cell biological steps for the evolution of the nucleus can be integrated into other frameworks as well and should have general validity concerning for example the evolution of the Ran gradient.

The minor comments I have are as follows:

p4: the description of the argument given in ref 18 is not accurate. That paper takes issue with Martin & Koonin's model (ref 17) wherein massive mitochondrion-derived group II intron infection of an archaeal host genome drives evolution of the nucleus. However, it argues that intron proliferation could not have happened before the origin of meiotic sex – no argument is made that such spread necessarily requires a nucleus.

p8: The argument for an open NE would be clearer if the relevant aspects of the model proposed by Devos et al. (ref 1) were summarised.

p9: If the author feels that an explanation of a torus needs to be given, then a reference work other than Wikipedia should be cited. The dictionary would suffice, though personally I think the reference can be removed altogether.

#### Author's response

I modified these parts.

### Reviewer's report 4

Patrick Forterre, Universite Paris Sud and Institut Pasteur, Paris, France

Gáspár Jékely has previously played an important role in pointing the importance of the Ran proteins in the origin of the eukaryotic nucleus. He convincingly shown that the eukaryotic nucleus evolved by modification of a pre-existing endomembrane system, strongly supporting the endogenous scenario for the origin of the nucleus. These scenarios contrast with the fashionable idea that the nucleus originated from an archaeal endosymbiont.

Considering that the rRNA and all informational proteins of eukaryotes are very divergent from bacterial ones, suggesting a long periods of evolution after the divergence between these two domains, the simplest endogeneous scenario for the origin for the nucleus is that the nucleus originated in a lineage of pre-eukaryotic cells, before the endosymbiosis that led to the presence of mitochondria in all modern eukaryotes. However, in the present manuscript, Gáspár Jékely adopts an alternative scenario, in which the eukaryotic nucleus originated relatively recently, i.e. between the mitochondrial endosymbiosis and the radiation of major eukaryotic divisions.

#### Author's response

I don't see why an inferred ancient divergence time – based on the assumption of a reliable rRNA molecular clock, which is far from being a safe one – should reveal the temporal order of the origin of the nucleus and the mitochondrium.

To support this assumption, Gáspár refers to a comparative genomic study of Koonin and colleagues (Mans et al., 2004) in which these authors, according to Gáspár, state that « *several components of the nuclear pore complex (NPC) and nuclear transport cycle are of probable alphaproteobacterial origin *» (Ref 10). This is somewhat confusing, suggesting that Koonin and colleagues have indeed shown (for instance by phylogenetic analysis) that components of the nuclear pore complex have a specific phylogenetic affinity for their alpha-proteobacterial homologues, which is not the case since. In fact, in the abstract of their paper, Koonin and colleagues only state that « *NPC and nuclear transport cycle are of probable endosymbiotic origin *». More importantly, this statement is only an hypothesis deduced from their favourite scenario for eukaryotic origin (fusion of an archaeon and the alpha-proteobacterium at the origin of eukaryotes), but it is absolutely not supported by the data. Indeed, NPC proteins have no homologues in bacteria, but only share conserved protein repeated domains with some widely distributed bacterial proteins. This is clear both from the data of Koonin and colleagues and from a paper published one year later by Bapteste and colleagues on the same topic. In their paper, Bapteste et al found 15 distantly related prokaryotic homologues of NCP, all with WD repeated domains and conclude that « *an ancient system of transmembrane transport that originated before the separation of the three domains was recruited during early eukaryotic evolution *» (Genome Biology, 6/R85, 2005).

#### Author's response

Your criticism agrees with that of Anthony Poole, and I changed this section and indicated that the data don't unequivocally support a symbiotic origin for NPC and nuclear transport proteins.

Considering the absence of homologues of the NCP proteins in Archaea or Bacteria, it is likely that the nucleus originated in a specific pre-eukaryotic lineage of organisms either ancestral or sister group of Archaea. As repeatedly pointed out by several authors, this also makes senses since eukaryotes have a capacity of phagocytosis that explains well why one of their ancestor was able to engulf the alpha proteobacterion at the origin of mitochondria. The large cells of the pre-eukaryotic lineage should have been already endowed with this properties (phagocytosis), which is unknown in Bacteria or Archaea.

#### Author's response

I totally agree with these points. However, the ability of phagocytosis does not necessarily mean that these cells also had a nucleus. I nevertheless acknowledge that one can make a strong case by asking how the integrity of the genome was maintained in a cell that already developed cytoskeletal dynamics and membrane traffic [4]. The answer to this could be that the cell was also able to regulate its cytoskeletal dynamics and the place of chromosomes in it, as it happens e.g. during open mitosis. Such regulation could in fact have been the first role of the emerging Ran system.

In the framework of the mixed origin of eukaryotes, Gáspár Jékely proposes an elegant explanation for the origin of the nucleus, suggesting that the nuclear membrane emerged progressively to prevent the formation of hybrid, less efficient, ribosomes, containing a mixture of proto-eukaryotic and bacterial ribosomal proteins.

#### Author's response

All theories on the origin of eukaryotes have to be mixed origin theories because at least the mitochondrium and a host cell have to come together to get to the eukaryotic cenancestor. I would like to emphasise though that my theory is not a chimeric or fusion theory that assumes that eukaryogenesis initiated when two prokaryotic lineages fused together. The chimerism in the title refers to extensive component mixing after mitochondrial symbiosis and, more specifically, chimeric ribosomes. I am an advocate of an initial phase of eukaryogenesis during which a dynamic endomembrane system, cytoskeleton and phagocytosis evolved [3,9,45]. The mitochondrium came later, and triggered the origin of the nucleus. The present model, however, is also consistent with strict chimeric theories, like the hydrogen hypothesis.

Indeed, he rightly notices that 23 genes encoding bacterial ribosomal proteins (from the endosymbiont) have been transferred to the chromosomes of ancestral eukaryotic cells before the divergence of the major eukaryotic lineages. He then suggests that these transfers corresponds to a neutral phase of eukaryotic evolution that occurred before the formation of the nucleus, leading to chimerism and reduced fitness. This in turn would have triggered a selective pressure to create a barrier separating the eukaryotic ribosome factory from the mitochondrial ribosome factory (the nuclear membrane).

However, in my opinion, there is a major default in this reasoning. The transfer of bacterial ribosomal proteins to the eukaryotic genome could not have not been both neutral and highly deleterious (reducing fitness) !! Accordingly, cells in which these transfers occurred would have been strongly counter-selected and would have simply disappeared. In fact, I think that Gáspár Jékely gives us a strong argument to think that transfer of bacterial genes from the mitochondrion to the eukaryotic genome occurred indeed after the formation of the nucleus. Otherwise, the transfer of mitochondrial ribosomal protein genes (being strongly counter-selected) would have simply never occurred in the first place.

#### Author's response

See my response to a similar concern raised by Martijn Huynen.

I agree with Gáspár Jékely that one should propose a positive selection pressure for the origin of the nucleus. In my opinion, such positive selection pressure could be the requirement for the cell to protect their genomes from the attack of incoming viruses. Modern viruses replicating in the cytoplasm of eukaryotic cells form nuclear-like structures by recruiting membranes of the endoplasmic reticulum to protect their genomes from the attack of their host. The complex modern nucleus can have originated progressively (possibly through some of the steps described in the Jékely's manuscript) in the interplay between viruses and cells, both eager to protect their genomes from the other by physical segregation. Later on, some viruses have finally managed to replicate inside the eukaryotic nucleus.

#### Author's response

I would of course be very interested in reading a detailed cell biological scenario on this interesting proposition.
